# Immune-Enhancing Effects of Taishan *Pinus massoniana* Pollen Polysaccharides on DNA Vaccine Expressing *Bordetella avium* ompA

**DOI:** 10.3389/fmicb.2016.00066

**Published:** 2016-02-02

**Authors:** Fujie Zhu, Xiao Liu, Zhenhong Sun, Cuilian Yu, Liping Liu, Shifa Yang, Bing Li, Kai Wei, Ruiliang Zhu

**Affiliations:** ^1^Laboratory of Animal Biological Products, College of Animal Science and Technology, Shandong Agricultural UniversityTaian, China; ^2^Analytic Laboratory, Institute of Preclinical Medicine, Taishan Medical CollegeTaian, China

**Keywords:** *Bordetella avium*, outer membrane protein A, DNA vaccine, *Pinus massoniana* pollen polysaccharides, adjuvant, chicken

## Abstract

*Bordetella avium* is the causative agent of bordetellosis, which remains to be the cause of severe losses in the turkey industry. Given the lack of vaccines that can provide good protection, developing a novel vaccine against *B. avium* infection is crucial. In this study, we constructed a eukaryotic expression plasmid, which expressed the outer membrane protein A (ompA) of *B. avium*, to prepare a *B. avium* recombinant ompA-DNA vaccine. Three concentrations (low, middle, and high) of Taishan *Pinus massoniana* pollen polysaccharides (TPPPS), a known immunomodulator, were used as adjuvants, and their immune conditioning effects on the developed DNA vaccine were examined. The pure ompA-DNA vaccine, Freund’s incomplete adjuvant ompA-DNA vaccine, and the empty plasmid served as the controls. The chickens in each group were separately inoculated with these vaccines three times at 1, 7, and 14 days old. Dynamic changes in antibody production, cytokine secretion, and lymphocyte count were then determined from 7 to 49 days after the first inoculation. Protective rates of the vaccines were also determined after the third inoculation. Results showed that the pure DNA vaccine obviously induced the production of antibodies, the secretion of cytokines, and the increase in CD^4+^ and CD^8+^ T lymphocyte counts in peripheral blood, as well as provided a protective rate of 50% to the *B. avium*-challenged chickens. The chickens inoculated with the TPPPS adjuvant ompA-DNA vaccine and Freund’s adjuvant ompA-DNA vaccine demonstrated higher levels of immune responses than those inoculated with pure ompA-DNA vaccine, whereas only the ompA-DNA vaccine with 200 mg/mL TPPPS completely protected the chickens against *B. avium* infection. These findings indicate that the *B. avium* ompA-DNA vaccine combined with TPPPS is a potentially effective *B. avium* vaccine.

## Introduction

*Bordetella avium* was first isolated from the respiratory tracts of turkeys in 1967 (Filion et al., 1967). *B. avium* is an acute infectious pathogen that demonstrates high horizontal infectivity and congenital transmissibility, and it mainly infects young chickens and turkeys (Beach et al., 2012). This pathogen has been found in many other avian species, including Muscovy ducks, domesticated geese, partridges, ostriches, cockatoos, macaws, parrot finches, and cockatiels (Hinz and Glunder, 1985; Raffel et al., 2002). Widespread dissemination of this pathogen both in wild and domesticated poultry was demonstrated by a serum prevalence survey (Raffel et al., 2002). Harrington et al. (2009) isolated a *B. avium* strain from patients with respiratory diseases and proved that *B. avium* is also an opportunistic pathogen in humans. The vaccines that are currently available to prevent *B. avium* disease are mainly live, temperature-sensitive, mutant vaccine, and whole-cell cephalosporins. Although the live vaccines and bacterins of *B. avium* can provide protection to at least 3-week-old turkeys, younger poultry respond poorly to vaccination (Rimler and Kunkle, 1998). Existing vaccines can also offer some protection against severe diseases; however, they do not limit the infection and spread of *B. avium* (Stockwell et al., 2011). Developing a novel vaccine to prevent *B. avium* infection is therefore necessary.

Since the first DNA vaccine was reported in 1990 (Wolff et al., 1990), the efficacy of DNA vaccines against infectious diseases have been demonstrated (Robinson et al., 1993; Sun et al., 2010; Borrego et al., 2011). Compared with the traditional inactivated, attenuated, and subunit vaccines, DNA vaccines exhibit more advantages by inducing a broad spectrum of cellular and humoral immune responses. DNA vaccine immunization can prolong the expression of antigens, as well as sustain their activity (Tang et al., 1992; Yankauckas et al., 1993), which represents a novel strategy to prevent or control some infectious diseases. Since DNA vaccines have become popular during the past two decades, this technology has brought both considerable excitement and disappointment. The magnitude of immune responses elicited by DNA vaccines are generally lower in humans and large animals than in small animals, as proven by a number of clinical trials (Laddy and Weiner, 2006). Therefore, further development of DNA vaccines has been limited by their relatively modest immunogenicity. To overcome this deficiency, several strategies have been proposed to enhance the efficacy of DNA vaccines. Among these, applying an adjuvant in vaccination is a good option to improve the immunogenicity of DNA vaccines.

Adjuvants are believed to activate the innate immune system, and thereby enhance the adaptive immune response to a simultaneously administered antigen (O’Hagan and De Gregorio, 2009; Mbow et al., 2010). Investigating novel plant compounds that modulate the immune system have become a promising field of research, particularly in searching for new-generation vaccine adjuvants (Licciardi and Underwood, 2011). Many polysaccharides, such as *Astragalus* polysaccharides, *Panax ginseng* polysaccharides, and *Ganoderma lucidum* polysaccharide F3, have been demonstrated their immunopotentiating function and adjuvant efficacy (Jiang et al., 2010; Lai et al., 2010; Ma et al., 2010). Notably, pine pollen, a kind of nutritional pollen, which is gloriously known as “King of Pollen” in China, has attracted our attention. The principal pine species in Mount Taishan region is *Pinus massoniana*, and the polysaccharides extracted from Taishan *Pinus massoniana* pollen has been studied in our laboratory since 2003, which has been found to be an effective adjuvant for inactivated and subunit vaccines (Wei et al., 2011; Cui et al., 2013). However, the use of Taishan *Pinus massoniana* pollen polysaccharides (TPPPS) as an adjuvant of DNA vaccines has not yet been investigated.

In the current study, to develop a novel *B. avium* vaccine, we constructed a recombinant plasmid expressing *B. avium* outer membrane protein A (ompA), which is an important component of the outer membrane protein and can induce high antibody titers as a protective antigen (Gentry-Weeks et al., 1992). To improve the immunogenicity of this DNA vaccine, TPPPS was used as an adjuvant for the DNA vaccine to examine its immune conditioning effects. We found that the recombinant ompA-DNA vaccine induced specific immune responses, although it incompletely protected the chickens against *B. avium* challenge. By contrast, the TPPPS adjuvant elevated the immune effects of the DNA vaccine to resist the *B. avium* challenge effectively.

## Materials and Methods

### Ethics Statement

The animal procedures used in this study were approved by the Animal Care and Use Committee of Shandong Agricultural University (Permit number: 20010510) and performed in accordance with the “Guidelines for Experimental Animals” of the Ministry of Science and Technology (Beijing, China).

### Bacterial Strains, Plasmids, and Cells

*Bordetella avium* LL strain was isolated in our laboratory in 2008 (Shandong, China). The strain was cultured and maintained in lysogeny broth agar and broth at 37°C. The eukaryotic expression plasmid pVAX1 was purchased from Invitrogen (Carlsbad, CA, USA). In addition, BHK-21 cells were maintained in minimum essential medium (MEM, GIBCO) that contained 10% fetal bovine serum (FBS, GIBCO) and 1% antibiotics (Invitrogen) at 37°C in a humidified atmosphere of 5% CO_2_.

### Construction of Recombinant ompA Plasmid

The primers were designed according to the ompA gene sequence of *B. avium* (GenBank accession number: M96550.1) using Primer 5.0. EcoR I and Xho I restriction enzyme sites were introduced into the respective 5′ termini (underlined): forward primer 5′-GCGAATTCCATGAACAAACCCTCCAA A-3′ and reverse primer 5′-CCGCTCGAGTTACTTGCGGCTA CCGACGATT-3′. The polymerase chain reaction (PCR) product was digested with EcoR I and Xho I and cloned into the pVAX1 vector. The resultant plasmid was confirmed by sequencing (Sunny, Shanghai) and then transformed into DH5α competent cells (*E. coli*) to amplify the yield.

### Transfection and Western Blot Analysis

Confluent BHK-21 cells were cultured in 6-well plates without antibiotics, and recombinant ompA plasmids (4 μg) were transfected into the BHK-21 cells using Lipofectamine (Invitrogen). The transfected cells were cultured in MEM with 1% FBS for 72 h. The expressed ompA in the cell culture supernatants was purified through nickel affinity chromatography according to a previous method (Asgarian-Omran et al., 2013) and then verified through Western blot analysis using an anti His-Tag monoclonal antibody (Cwbio, China; Temple et al., 2010).

### Vaccine Preparation

Taishan *Pinus massoniana* pollen polysaccharides was prepared in our laboratory through water extraction and ethanol precipitation (Wei et al., 2011). The recombinant plasmids in *E. coli* DH5α were extracted using EndoFree Plasmid Giga Kit (Qiagen). Plasmid concentration was determined using NanoDrop (Thermo). The recombinant plasmids were then mixed with TPPPS or Freund’s incomplete adjuvant (Solarbio, Beijing) at a ratio of 1:1 up to a final concentration of 250 μg/mL. The contents of TPPPS were set at three doses, namely, 50 (low), 100 (moderate), and 200 (high) mg/mL, in three separate TPPPS adjuvant vaccines. The recombinant plasmids mixed with three doses of TPPPS and Freund’s adjuvant were separately prepared to obtain the corresponding adjuvant DNA vaccines.

### Animal Experiment

A total of 224 one-day-old specific pathogen-free white leghorn chickens (male; Spirax Ferrer Poultry Co., Ltd, Jinan) were randomly separated into seven sterilized isolators, and each isolator contained a group of 32 chickens (groups I–VII). The ambient conditions were set to 20–25°C and 30–40% relative humidity, and the air entering the isolators was filtered.

The chickens in groups I–VII were intramuscularly injected with 0.4 mL low, moderate, and high doses TPPPS adjuvant ompA-DNA vaccines, Freund’s adjuvant ompA-DNA vaccine, pure ompA-DNA vaccine, empty plasmid, and phosphate buffered saline (PBS), respectively, at 1, 7, and 14 days old. Groups I–VII were named OmpA-TPPPS (L), OmpA-TPPPS (M), OmpA-TPPPS (H), OmpA-Freund, OmpA, Empty plasmid, and Mock, respectively. At 7, 14, 21, 28, 35, 42, and 49 days post the first inoculation (dpi), three chickens from each group were selected randomly to determine the antibody, IFN-γ, IL-2, and IL-4 concentrations in serum, as well as the CD^4+^ and CD^8+^ T lymphocytes counts in peripheral blood. The animals were starved for 12 h before sampling.

One week after the third inoculation (21 dpi), 20 chickens in each group (including nine sampled and 11 unsampled chickens) were placed into a new isolator and then challenged intraperitoneally with 10 median lethal dose (LD_50_) *B. avium* LL strain. Clinical symptoms, including labored breathing, sneezing, and oculonasal discharges, were monitored for 7 days after challenge. The protective rate in each group were calculated according to the following formulas:

$$\mathrm{Pr}otective\,\;rate\left(\%\right)=\frac{No.\,\;of\,\;\,chickens\,\;without\,\;clinical\,\;symptoms}{Total\,\;\,No.\times 100\%}$$

### Detection of Serum Antibody Titers, IFN-γ, IL-2, and IL-4

Three serum samples from each group were randomly collected during sampling. The serum antibody titers were detected via enzyme-linked immunosorbent assay (ELISA) as described previously (Li et al., 2012). In addition, the cytokines of IFN-γ, IL-2, and IL-4 were detected using the chicken IFN-γ, IL-2, and IL-4 ELISA kits (Langdon Bio-technology Co., Ltd, Shanghai), respectively. The absorbance at 450 nm was measured using an ELISA reader.

### Detection of CD^4+^ and CD^8+^ T Lymphocytes in Peripheral Blood

Fresh, anticoagulant-treated peripheral blood samples from three chickens in each group were randomly collected and separately mixed with an equivalent volume of PBS. Then, 2 mL of the mixture was added into 3 mL lymphocyte separation medium (Solarbio, China), which was centrifuged at 2000 rpm for 15 min. The lymphocytes were harvested and washed twice with PBS. CD^4+^ dye (10 μL, Mouse Anti-chicken CD4-FITC, Cat: 8210-02, Lot: H4809-MM62, USA) and CD^8+^ dye (10 μL, Mouse Anti-chicken CD8-RPE, Cat: 8220-09, Lot: G149-VJ39K, USA) were added into 50 μL lymphocyte suspension. The suspension was incubated at 4°C for 20 min. The percentages of CD^4+^ and CD^8+^ T lymphocytes were detected via flow cytometry (Guaga Easy Cyte Mini, USA).

### Statistical Analysis

Data were presented as mean ± standard deviation (SD), and Duncan’s multiple-range test was performed to analyze the differences among groups using SPSS 17.0 software. A *P*-value of <0.05 was considered statistically significant.

## Results

### Construction and Expression of Recombinant ompA Plasmid

**FIGURE 1:**
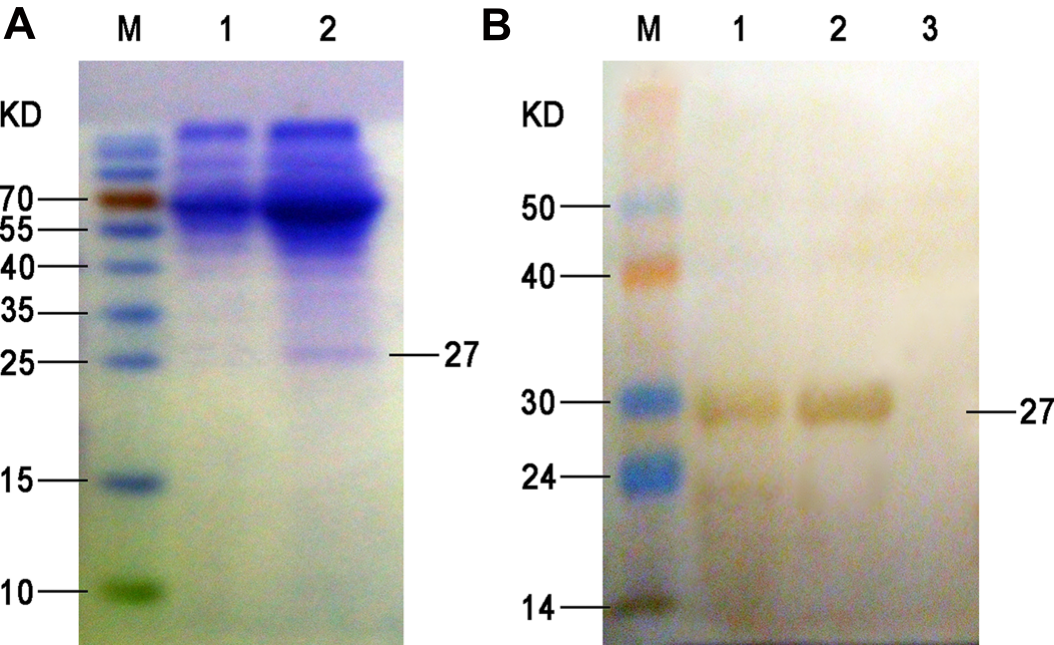
**SDS-PAGE (A) and Western blot (B) of the expressed ompA in the supernatant. (A)** M, protein molecular weight markers; lane 1, proteins isolated from the culture supernatant of BHK-21 cells at 72 h after empty plasmid transfection (negative control); lane 2, proteins isolated from the culture supernatant of BHK-21 cells at 72 h after pVAX1-ompA plasmid transfection. **(B)** M, PageRuler prestained protein ladder; 1, proteins isolated from the culture supernatant of BHK-21 cells at 72 h after pVAX1-ompA plasmid transfection; 2, purified recombinant protein from eluted fraction; 3, proteins isolated from the culture supernatant of BHK-21 cells at 72 h after empty plasmid transfection (negative control).

Firstly, the ompA gene of *B. avium* was amplified by PCR, and agarose gel electrophoresis showed that the size of the amplified ompA PCR products was 597 bp, which was consistent with the expected ompA fragment size. Then the PCR products were cloned into the pVAX1 vector, and the recombinant plasmid was verified through restriction digestion and sequencing (data not shown). After transfection into the BHK-21 cells, the target recombinant protein was purified via nickel affinity chromatography based on the presence of 6× His tag in the fusion protein. The contaminant proteins were washed with buffer with 20 mM imidazole, and the target protein was eluted with buffer that contained 0.5 M imidazole. The protein fractions were analyzed via SDS-PAGE and Western blot analysis, and the results showed that a single protein band with an apparent molecular weight of 27 kDa was obtained after purification (**Figure 1**).

### Changes in Serum Antibody Titers

**FIGURE 2:**
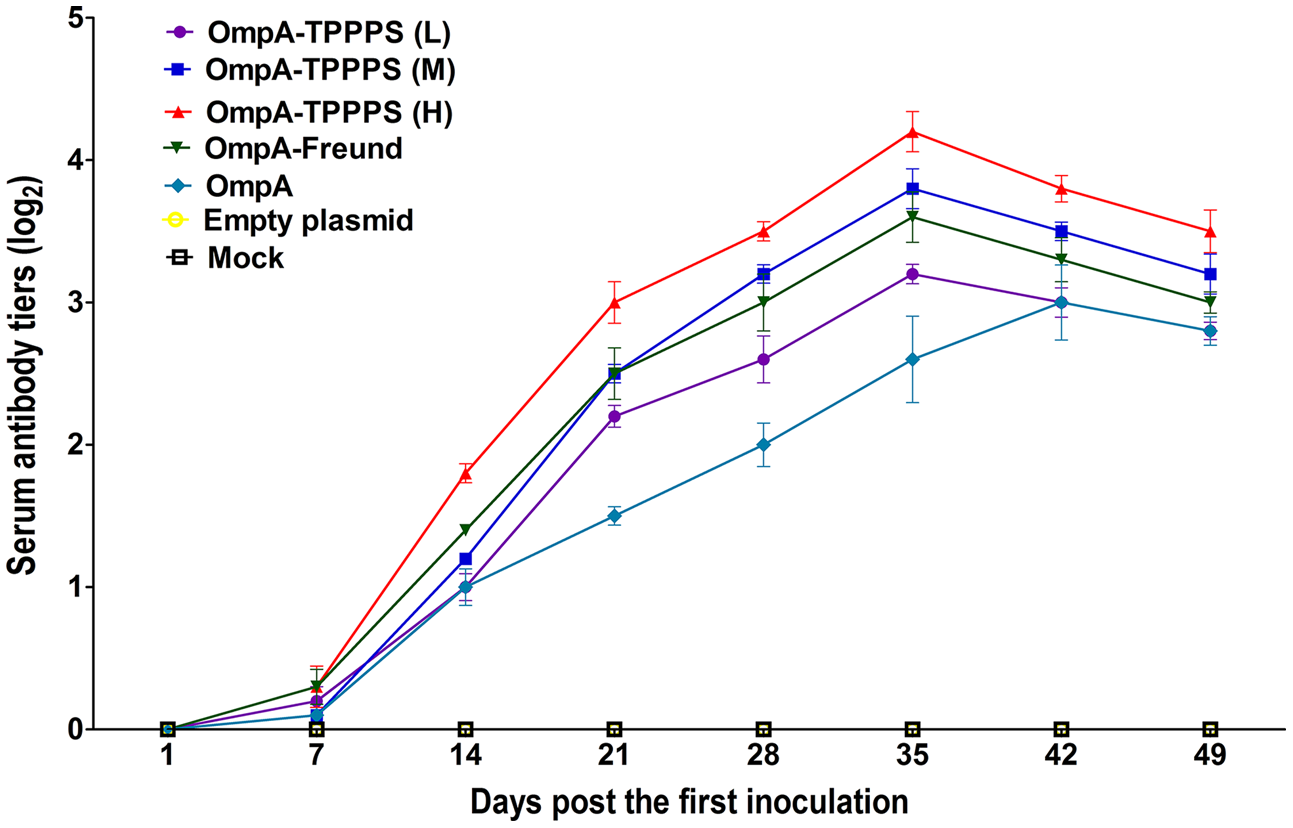
**Changes in the serum antibody titers of the chickens inoculated with vaccines.** Chickens in the seven groups were inoculated with 50 (purple), 100 (blue), and 200 (red) mg/mL of TPPPS adjuvant ompA-DNA vaccines, Freund’s adjuvant ompA-DNA vaccine (green), pure ompA-DNA vaccine (cyan), empty plasmid (yellow), and PBS (black), respectively, at 1, 7, and 14 days old. Serum was collected at 7, 14, 21, 28, 35, 42, and 49 dpi. The antibody titers were then determined via indirect ELISA. All values shown are the means ± SD of three independent experiments.

The levels of antibody induced by vaccination are the key to examining the effects of vaccines. The dynamic changes in the serum antibody titers in each group are presented in **Figure 2**. At 14 dpi, the serum antibody titers in groups OmpA-TPPPS (L), (M), (H), OmpA-Freund, and OmpA increased and were significantly higher than those in groups Empty plasmid and Mock (*P* < 0.05). Notably, the serum antibody titers in group OmpA-TPPPS (H) were significantly higher than those in other groups (*P* < 0.05) at 14–49 dpi. At 35 dpi, the serum antibody titers in groups OmpA-TPPPS (L), (M), (H), and OmpA-Freund reached their peak value and were higher than those in group OmpA. However, the antibody titers in group OmpA peaked at 42 dpi.

### Changes in Serum IFN-γ, IL-2, and IL-4

**FIGURE 3:**
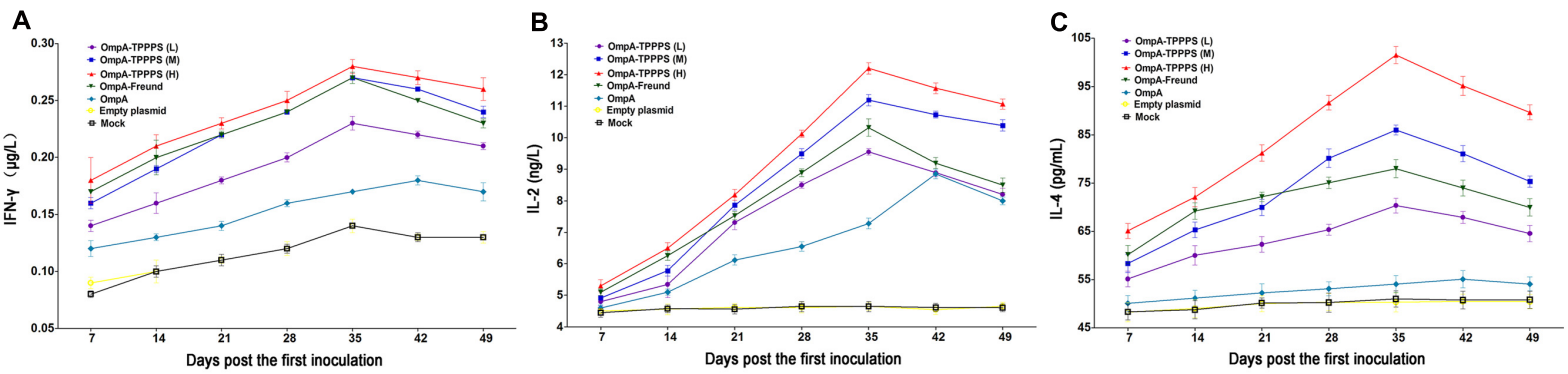
**Changes in cytokines in the chickens inoculated with vaccines.** Chickens in the seven groups were inoculated with 50 (purple), 100 (blue), and 200 (red) mg/mL of TPPPS adjuvant ompA-DNA vaccines, Freund’s adjuvant ompA-DNA vaccine (green), pure ompA-DNA vaccine (cyan), empty plasmid (yellow), and PBS (black), respectively, at 1, 7, and 14 days old. Serum was collected at 7, 14, 21, 28, 35, 42, and 49 dpi. IFN-γ **(A)**, IL-2 **(B)**, and IL-4 **(C)** were detected using the chicken IFN-γ, IL-2, and IL-4 ELISA kits. All values shown are the means ± SD of three independent experiments.

Cytokines are crucial in fighting off infections and are involved in immune responses (Lowry, 1993). The results of serum IFN-γ, IL-2, and IL-4 concentrations are shown in **Figure 3**. As shown in **Figures 3A,B**, the serum IFN-γ and IL-2 concentrations were significantly higher in group OmpA than in groups Empty plasmid and Mock (*P* < 0.05). Moreover, the concentrations of these two cytokines were significantly higher in groups OmpA-TPPPS (L), (M), (H), and OmpA-Freund than in group OmpA (*P* < 0.05), and these two indicators were significantly higher in group OmpA-TPPPS (H) than in other groups (*P* < 0.05). As shown in **Figure 3C**, the concentrations of serum IL-4 in group OmpA-TPPPS (H) were significantly higher than in other groups (*P* < 0.05). The overall IL-4 concentrations in groups OmpA-TPPPS (L), (M), (H), and OmpA-Freund were also significantly higher than in groups OmpA, Empty plasmid, and Mock (*P* < 0.05). A comparison between group OmpA and groups Empty plasmid and Mock indicated that the serum IL-4 concentrations were higher in group OmpA than in groups Empty plasmid and Mock, although the disparity was insignificant (*P* > 0.05).

### Changes in CD^4+^ and CD^8+^ T Lymphocyte Counts in the Peripheral Blood

CD^4+^ and CD^8+^ T lymphocyte counts directly reflect immune function in animals (Torti et al., 2012). **Tables 1** and **2** show the percentages of CD^4+^ and CD^8+^ T lymphocytes in the peripheral blood, respectively, where the percentages of CD^4+^ and CD^8+^ T lymphocytes were significantly higher in group OmpA than in groups Empty plasmid and Mock (*P* < 0.05). Moreover, the percentages of CD^4+^ and CD^8+^ T lymphocytes in groups OmpA-TPPPS (L), (M), (H), and OmpA-Freund were significantly higher than those in group OmpA (*P* < 0.05). Notably, The percentages of CD^4+^ and CD^8+^ T lymphocytes were also significantly higher in group OmpA-TPPPS (H) than in other groups (*P* < 0.05).

**Table 1:** Changes of CD^4+^ T lymphocyte counts in peripheral blood.

Group^∗^	Days post the first inoculation (d)^#^						
	7	14	21	28	35	42	49
OmpA-TPPPS (L)	7.26 ± 0.83^a^	8.81 ± 0.61^a^	11.33 ± 0.63^a^	13.25 ± 1.13^a^	14.49 ± 0.42^a^	15.76 ± 0.35^a^	14.67 ± 1.02^a^
OmpA-TPPPS (M)	9.11 ± 0.93^b^	12.44 ± 0.81^b^	15.84 ± 0.78^b^	17.45 ± 0.99^b^	18.33 ± 0.79^b^	20.26 ± 0.85^b^	17.65 ± 0.74^b^
OmpA-TPPPS (H)	11.70 ± 0.06^c^	15.38 ± 0.45^c^	18.83 ± 0.47^c^	22.04 ± 0.49^c^	24.29 ± 0.67^c^	27.98 ± 1.05^c^	25.32 ± 0.80^c^
OmpA-Freund	9.84 ± 0.41^b^	13.00 ± 0.80^b^	15.59 ± 0.79^b^	18.00 ± 0.90^b^	18.66 ± 0.11^b^	19.59 ± 0.62^b^	17.00 ± 0.80^b^
OmpA	5.00 ± 0.90^d^	6.26 ± 0.86^d^	8.18 ± 0.75^d^	10.36 ± 0.79^d^	11.28 ± 0.10^d^	12.30 ± 0.10^d^	10.01 ± 0.69^d^
Empty plasmid	4.40 ± 0.60^d^	5.00 ± 0.10^e^	6.00 ± 0.90^e^	7.37 ± 0.15^e^	6.99 ± 0.55^e^	7.24 ± 0.70^e^	6.72 ± 0.24^e^
Mock	4.30 ± 0.30^d^	5.00 ± 0.40^e^	5.00 ± 0.50^e^	7.41 ± 0.71^e^	6.52 ± 0.42^e^	6.38 ± 0.13^e^	6.44 ± 0.11^e^

*^∗^Group names represent that chickens in these groups were inoculated with 50, 100, and 200 mg/mL of TPPPS adjuvant ompA-DNA vaccines, Freund’s adjuvant ompA-DNA vaccine, pure ompA vaccine, empty plasmid, and PBS, respectively.*

*^#^CD^4+^ T lymphocyte counts in the same column are compared by Duncan’s multiple-range tests. Different lowercase letter superscripts indicate significant differences (P<0.05). Data are expressed as mean percentage ± SD.*

**Table 2:** Changes of CD^8+^ T lymphocyte counts in peripheral blood.

Group^∗^	Days post the first inoculation (d)^#^						
	7	14	21	28	35	42	49
OmpA-TPPPS (L)	5.56 ± 0.86^a^	7.23 ± 0.21^a^	9.26 ± 0.69^a^	10.26 ± 0.55^a^	11.18 ± 0.33^a^	13.31 ± 0.38^a^	12.01 ± 0.12^a^
OmpA-TPPPS (M)	7.03 ± 0.20^b^	8.00 ± 0.55^a^	10.60 ± 0.35^b^	12.00 ± 0.50^b^	14.00 ± 0.70^b^	16.03 ± 0.35^b^	14.87 ± 0.61^b^
OmpA-TPPPS (H)	8.03 ± 0.45^c^	10.00 ± 0.40^b^	12.29 ± 0.27^c^	14.10 ± 0.38^c^	16.37 ± 0.75^c^	17.94 ± 0.83^c^	16.82 ± 0.87^c^
OmpA-Freund	7.97 ± 0.25^c^	9.00 ± 0.60^c^	11.00 ± 0.90^b^	12.93 ± 0.40^b^	14.00 ± 0.30^b^	15.00 ± 0.50^d^	13.10 ± 0.10^d^
OmpA	4.99 ± 0.02^a^	5.98 ± 0.90^d^	6.66 ± 0.87^d^	7.95 ± 0.83^d^	9.00 ± 0.50^d^	11.00 ± 0.60^e^	8.02 ± 0.67^e^
Empty plasmid	4.18 ± 0.35^d^	4.96 ± 0.44^e^	5.39 ± 0.30^e^	5.96 ± 0.48^e^	5.75 ± 0.40^e^	5.38 ± 0.39^f^	5.65 ± 0.48^f^
Mock	4.23 ± 0.32^d^	4.70 ± 0.34^e^	5.42 ± 0.42^e^	5.94 ± 0.48^e^	5.78 ± 0.36^e^	5.58 ± 0.38^f^	5.58 ± 0.02^f^

*^∗^Group names represent that chickens in these groups were inoculated with 50, 100, and 200 mg/mL of TPPPS adjuvant ompA-DNA vaccines, Freund’s adjuvant ompA-DNA vaccine, pure ompA vaccine, empty plasmid, and PBS, respectively.*

*^#^CD^8+^ T lymphocyte counts in the same column are compared by Duncan’s multiple-range tests. Different lowercase letter superscripts indicate significant differences (P < 0.05). Data are expressed as mean percentage ± SD.*

### Protective Effects of the Vaccines

**FIGURE 4:**
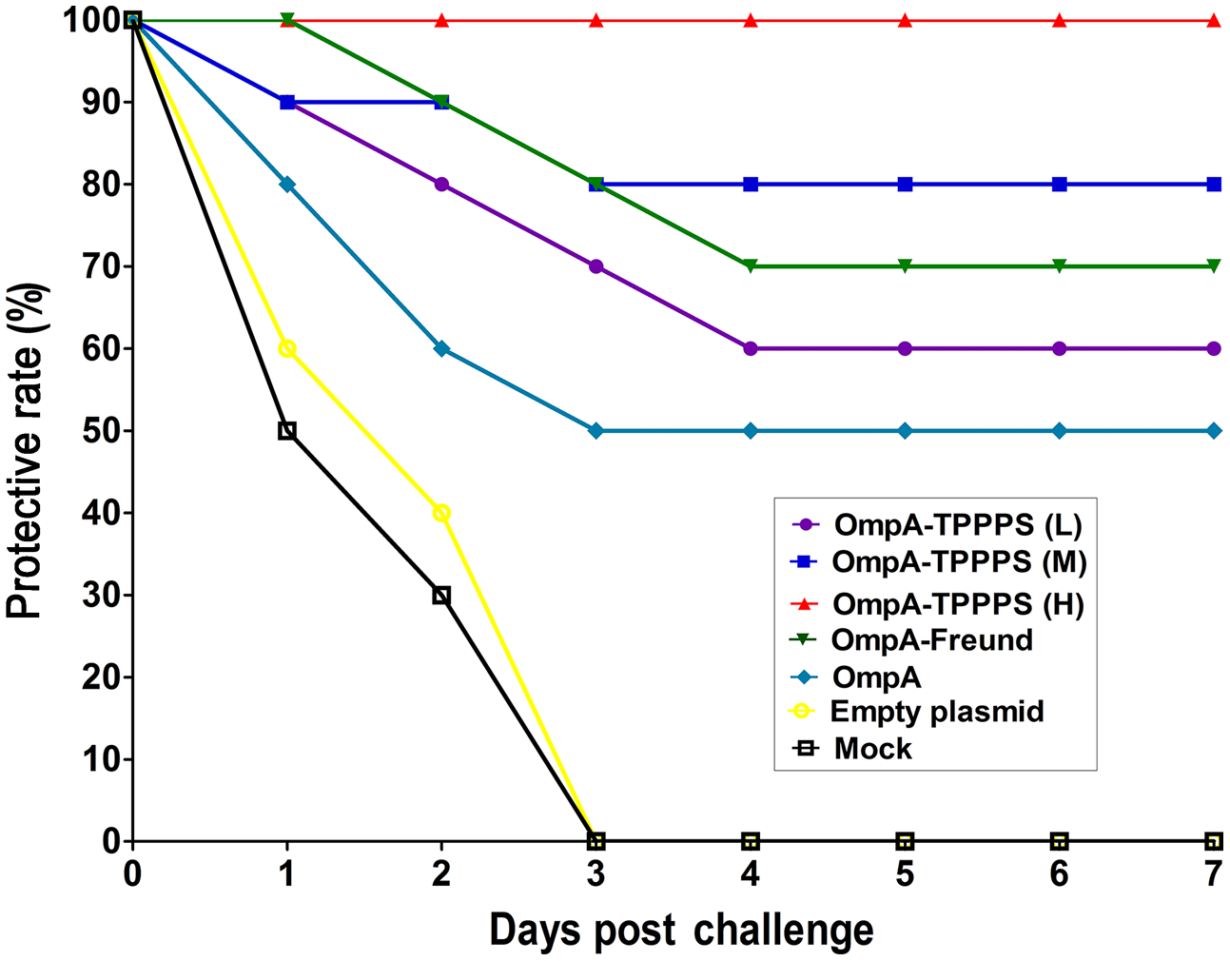
**Protective rates of *B. avium*-challenged chickens.** Chickens in the seven groups were inoculated with 50 (purple), 100 (blue), and 200 (red) mg/mL of TPPPS adjuvant ompA-DNA vaccines, Freund’s adjuvant ompA-DNA vaccine (green), pure ompA-DNA vaccine (cyan), empty plasmid (yellow), and PBS (black), respectively, at 1, 7, and 14 days old. One week after the third inoculation, 20 chickens from each group were challenged intraperitoneally with 10 LD_50_ *B. avium* LL strain. Clinical symptoms, including labored breathing, sneezing, and oculonasal discharges, were monitored for seven successive days after the challenge.

To evaluate the protective effects of the ompA-DNA vaccine against *B. avium* infection, we challenged 20 chickens from each group with 10 LD_50_ *B. avium* LL strain at 1 week post the third inoculation. Clinical symptoms were monitored daily, and the protective rates of different groups were calculated as described in the section “Materials and Methods” (**Figure 4**). The result showed that the morbidity was significantly lower in chickens inoculated with the ompA-DNA vaccine than in chickens in the groups Empty plasmid and Mock (*P* < 0.05). In addition, the protective rate in group OmpA-TPPPS (H) was significantly higher than those in other groups (*P* < 0.05). These data demonstrated that the pure ompA-DNA vaccine could protect the chickens against *B. avium* infection at a rate of 50%, and the ompA-DNA vaccine with 200 mg/mL TPPPS completely protected the chickens against *B. avium* infection.

## Discussion

*Bordetella avium* is the causative agent of a highly contagious upper respiratory disease in young poultry, and existing vaccines provide limited protection only. In this study, we constructed a recombinant plasmid that expressed *B. avium* ompA, which was used to prepare a novel *B. avium* DNA vaccine. In addition, TPPPS was used as the adjuvant of this DNA vaccine, and its immune conditioning effects were examined. We determined that the pure ompA-DNA vaccine induced specific immune responses and protected the chickens, which displayed a 50% protective rate. By contrast, the TPPPS adjuvant ompA-DNA vaccine elicited remarkably higher levels of serum antibody titers, cytokines, and T lymphocyte counts, as well as caused higher protective rates among the chickens.

DNA vaccine, also known as nucleic acid vaccine, is a new research field that has been developed in the research of gene therapy in recent years. DNA vaccine can express antigen protein via eukaryotic expression pattern when it is absorbed by the host cells; the sustained expression can induce a strong and lasting immune response in vertebrates. In this study, the natural ompA of *B. avium* is a prokaryotic protein, and its molecular structure may become more complex when it is expressed in the eukaryotic expression system. Thus, some epitopes may be altered or hidden. However, our results have proven that the ompA expressed in host cells also provided a certain immune protection, thereby indicating that it retained certain epitopes and immunogenicity of natural ompA. Moreover, we found that the addition of TPPPS adjuvant remarkably improved the immune protective effects of ompA-DNA vaccine. This result further verified the specific antigenicity of ompA and implied the immunoregulatory activity of TPPPS.

DNA vaccine offers distinctive advantages compared with traditional vaccines. This type of vaccine promotes the expression of specific antigens in host cells and elicits both humoral and cellular immune responses. In addition, it is easy to produce, relatively inexpensive, homogeneous and heat stable (Belakova et al., 2007). However, given that the immune response of DNA vaccines depends on several parameters, such as the selection of the candidate antigen, vaccine vector, adjuvant type, and administration route (Oran and Robinson, 2003; Perrie et al., 2003; Sasaki et al., 2003; Liu, 2011), all of these factors may possibly influence the effects of vaccination. The safety of using vaccine vectors in animals is a primary consideration. This study employed the pVAX1 plasmid, the use of which was approved by the US Food and Drug Administration (“Points to Consider on Plasmid DNA Vaccines for Preventive Infectious Disease Indications,” published on December 22, 1996, Docket No. 96N-0400). To date, the pVAX1 plasmid is the safest vector in DNA vaccine, because it demonstrates minimal possibility of chromosomal integration and is less likely to elicit allergic responses. Studies have reported that pure pVAX1 plasmid does not exert any negative effect on animals and does not interfere with the efficacy of DNA vaccines (Song et al., 2009, 2010), which is consistent with our results. Moreover, we chose traditional intramuscular injection as the inoculation method in our animal experiment. Some studies, however, have shown that inoculation via electroporation can enhance plasmid uptake and immune responses (Bodles-Brakhop and Draghia-Akli, 2008). Many novel DNA delivery systems have also been invented, including gene gun and synthetic delivery systems. Thus, further studies must investigate the effects of the ompA-DNA vaccine using novel delivery systems.

Two types of T helper (Th) clones (Th1 and Th2 cells) differ in cytokine secretion patterns and in other functions (Mosmann et al., 1986). The major cytokines associated with the Th1 cell subsets are IL-2, TNF-α, and IFN-γ, which can enhance Tc cellular cytotoxicity and cell-mediated immune responses. The Th2 cell subsets secrete IL-4, IL-5, and IL-10, and these cytokines mainly promote antibody production and mediate humoral immune responses. We observed that the ompA-DNA vaccine significantly enhanced the production of IFN-γ and IL-2, but not IL-4, which indicated that the ompA-DNA vaccine mainly induced Th1 immune response. In antibacterial defense, these Th1 cytokines may enhance the proliferation and differentiation of lymphocytes and bactericidal activities of the innate immune cells, such as nature killer cells and macrophages.

Adjuvants are widely applied to improve the immunogenicity of subunit virus-like particles and DNA vaccines (Buonaguro et al., 2011). Various plant polysaccharides, such as *Astragalus*, *Panax ginseng*, and Taishan *Robinia pseudoacacia* polysaccharides, cannot only activate immunocytes but can also improve cytokine secretion (Jiang et al., 2010; Ma et al., 2010; Zhang et al., 2014). They also activate complement and reticuloendothelial systems, thereby regulating the immunologic function of organisms (Currier et al., 2003; Lai et al., 2010). Moreover, plant polysaccharides are typically less immunogenic, non-toxic, and biodegradable, and thus, are considered novel natural immune potentiator. Our previous studies have revealed that TPPPS can significantly strengthen immune response and enhance the effects of vaccines (Wei et al., 2011; Cui et al., 2013). In addition, TPPPS is water-soluble and exhibits high hydrophilicity and viscidity, which are suitable properties for an adjuvant (Wei et al., 2011). The present study showed that the chickens inoculated with the TPPPS adjuvant ompA-DNA vaccine displayed higher immunity and protective rate than those inoculated with the pure ompA-DNA vaccine. Notably, the 200 mg/mL TPPPS exhibited the best capability to improve the effects of the ompA-DNA vaccine. TPPPS also shortened the time before antibody levels peaked. Therefore, TPPPS is a potential vaccine adjuvant.

## Conclusion

This study demonstrated that the ompA-DNA vaccine induced both humoral and cellular immune responses (particularly the Th1 type). TPPPS, which was used as the adjuvant, effectively improved the immunogenicity of DNA vaccine, including antibody production, cytokine secretion, lymphocyte differentiation, and protective rate. The results of this study indicated the potential of the *B. avium* ompA-DNA vaccine combined with TPPPS adjuvant in preventing *B. avium* infection.

## Author Contributions

RZ, KW, FZ, XL, and ZS designed research; FZ, XL, CY, LL, SY, and BL performed research; FZ, XL, ZS, KW, and RZ analyzed data; FZ, XL, KW, and RZ wrote the paper.

## Conflict of Interest Statement

The authors declare that the research was conducted in the absence of any commercial or financial relationships that could be construed as a potential conflict of interest.
